# Nomogram for predicting outcomes in elderly women with mucinous breast cancer: A retrospective study combined with external validation in southwest China

**DOI:** 10.1002/cnr2.2112

**Published:** 2024-07-25

**Authors:** Zhaoxia Zhang, Chenghao Zhanghuang, Qian Cai, Guangye Song, Quan Wang, Yue Tang, Hongbo Li

**Affiliations:** ^1^ Department of Urology; Chongqing Key Laboratory of Children Urogenital Development and Tissue Engineering; Chongqing Key Laboratory of Pediatrics; Ministry of Education Key Laboratory of Child Development and Disorders; National Clinical Research Center for Child Health and Disorders; China International Science and Technology Cooperation base of Child development and Critical Disorders Children's Hospital of Chongqing Medical University Chongqing People's Republic of China; ^2^ Department of Cardiothoracic Surgery, Ministry of Education Key Laboratory of Child Development and Disorders, National Clinical Research Center for Child Health and Disorders, China International Science and Technology Cooperation Base of Child Development and Critical Disorders Children's Hospital of Chongqing Medical University Chongqing China; ^3^ Department of Urology Affiliated Hospital of Yunnan University (The Second People's Hospital of Yunnan Province, Ophthalmic Hospital of Yunnan Province) Kunming People's Republic of China; ^4^ Department of Surgery Kunming Hospital of Traditional Chinese Medicine Kunming People's Republic of China

**Keywords:** CSS, elderly, mucinous breast cancer, nomogram, OS, SEER

## Abstract

**Objective:**

Mucinous breast cancer (MBC) is a kind of breast cancer (BC), which is rare in clinic, mainly for women, because of the low incidence rate, so there is no unified standard treatment protocol. Elderly patients have a poor prognosis due to their combined comorbidities. This study aims to investigate the effect of surgery and chemoradiotherapy on the prognosis of elderly female MBC patients and construct nomograms for predicting the OS and CSS in elderly female MBC patients.

**Methods:**

Data for female MBC patients over 65 years are obtained from the Surveillance, Epidemiology and End Results (SEER) database, patients were divided into two groups: the training set and the validation set. External validation data of the prediction model were provided by Kunming Hospital of Traditional Chinese Medicine. We used Cox regression modeling, which was used to identify independent risk factors affecting patient prognosis. After avoiding confounding bias according to the multifactorial Cox regression model, we used these screened statistically significant results to construct column‐line plots. The performance of the model was tested using the consistency index (c‐index), the calibration curve, and the area under the operating characteristic curve of the receiver (AUC). Subsequently, we used decision curve analysis (DCA) to examine the potential clinical value of our nomograms.

**Results:**

A total of 8103 elderly MBC female patients were extracted from the database SEER and were assigned to the training and validation set, randomly. A total of 83 patients from Kunming Hospital of Traditional Chinese Medicine were used in the external verification set. After multifactorial Cox regression analysis, we found that age, race, T‐stage, M‐stage, surgical approach, radiotherapy, and tumor size were independent risk factors for OS in elderly MBC patients. Similarly, independent risk factors of CSS included age, marital status, N stage, M stage, surgical approach, chemotherapy, and tumor size. The C‐index for the OS training, validation, and external verification set were 0.731 (95%CI 0.715–0.747), 0.738 (95%CI 0.724–0.752), and 0.809 (95%CI 0.731–0.8874). The C‐index of the training set, the validation set, and external verification set for CSS were 0.786 (95%CI 0.747–0.825), 0.776 (95%CI 0.737–0.815), and 0.84 (95%CI0.754–0.926), respectively. The AUC, calibration curves and DCA also showed good accuracy.

**Conclusions:**

In this study, we construct a new nomogram to predict the prognosis of elderly patients with MBC. The nomograms have undergone internal and external validation and have been confirmed to have good clinical applicability. At the same time, we found that for elderly female MBC patients, surgery and radiotherapy significantly benefit their survival, but chemotherapy is not conducive to patient survival.

## 1 INTRODUCTION

Breast cancer (BC) is one of the most common type of cancers worldwide and can lead to a large number of cancer‐related deaths.^1^ In the United States, about 284 200 new cases of BC in 2021, among them, 281 550 are women, and total of 44 130 deaths, with 43 600 women.^2^ This shows that female patients dominate breast cancer. Mucinous breast cancer (MBC) is a clinically rare pathologic type of BC that accounts for approximately 1% ~ 6% of primary BC and is more common in older postmenopausal women.^3^ It has a better prognosis compared with other breast malignancies (such as ductal or lobular variants).^4^ The overall prognosis of MBC is favorable. The 5‐year overall survival rate (OS) of MBC is reported to be 95% to 98.9%, and the 10‐year overall survival rate exceeds 90%.^5^, ^6^ However due to the low incidence and clinical rarity, there is no uniform standard treatment regimen for MBC patients.

Results derived from single‐center clinical studies are prone to bias due to the low incidence of MBC. The Surveillance, Epidemiology, and End Results (SEER) is a National Cancer Institute database that records the incidence, mortality, and morbidity of millions of cancer patients in selected states and counties in the United States (US), which has the advantage of being updated in real time and having a large number of cases, covering more than 30% of the US population. SEER database‐based studies often represent population‐based extensive data studies, and the results are often more reliable than single‐center studies. The prognosis of MBC is often influenced by multiple factors. However, previous oncological prognosis was usually assessed by performing a conventional TNM staging system. Because the TNM staging system contains too few variables to predict some prognostic factors for patients in actual situations, it is difficult to propose personalized treatment options for each patient. The nomogram is a computer‐based predictive model that is presented graphically and is exceptionally friendly to users and can help clinicians to make personalized predictions.^7^ Currently, the investigators have developed many nomograms of BC based on the data of the SEER database, including nomogram predicting the OS of inflammatory BC patients, nomogram predicting the factors influencing complete pathological response after neoadjuvant chemotherapy of BC patients, and nomogram of personalized radiotherapy of BC patients.^8^, ^9^, ^10^ Zhu et al. developed a nomogram that could predict OS in MBC patients, suggesting that age, ethnicity, T stage, M stage, surgery, and radiotherapy were independent risk factors for OS in MBC patients.^11^ The nomogram developed by Fu et al. Can predicts the CSS of MBC patients with early stage.^12^ However, there are no current studies to construct both the nomograms of OS and CSS in MBC patients and explore the differences between the factors influencing them.

The MBC patients were mostly female, and the vast majority of MBC occurred in postmenopausal women. A single‐center study conducted by Emilia Marrazzo et al. showed that the median age of MBC patients was 64.4 years.^13^ In addition, the WHO and Medicare define older adults as over 65 years old.^14^, ^15^ However, no corresponding nomogram has been developed for elderly female MBC patients. Moreover, their non‐cancer‐specific death may lead to a low overall OS due to comorbidities, making OS and CSS different. Therefore, it is more important to construct a nomogram predicting OS and CSS in elderly MBC patients. Therefore, this study aims to construct nomograms to predict OS and CSS of elderly female MBC patients and further analyze the effects of surgical methods and chemoradiotherapy on OS and CSS through the KM curve in order to provide more reasonable guidance for the treatment of elderly female MBC patients.

## 2 METHODS

### 2.1 Dataset Description and Preprocessing

Data from patients diagnosed with BC during the period 2000–2018 were extracted from the SEER database. The target population of this study was female patients diagnosed with MBC aged over 65. This study followed the research guidelines published by the SEER database.

**FIGURE 1:**
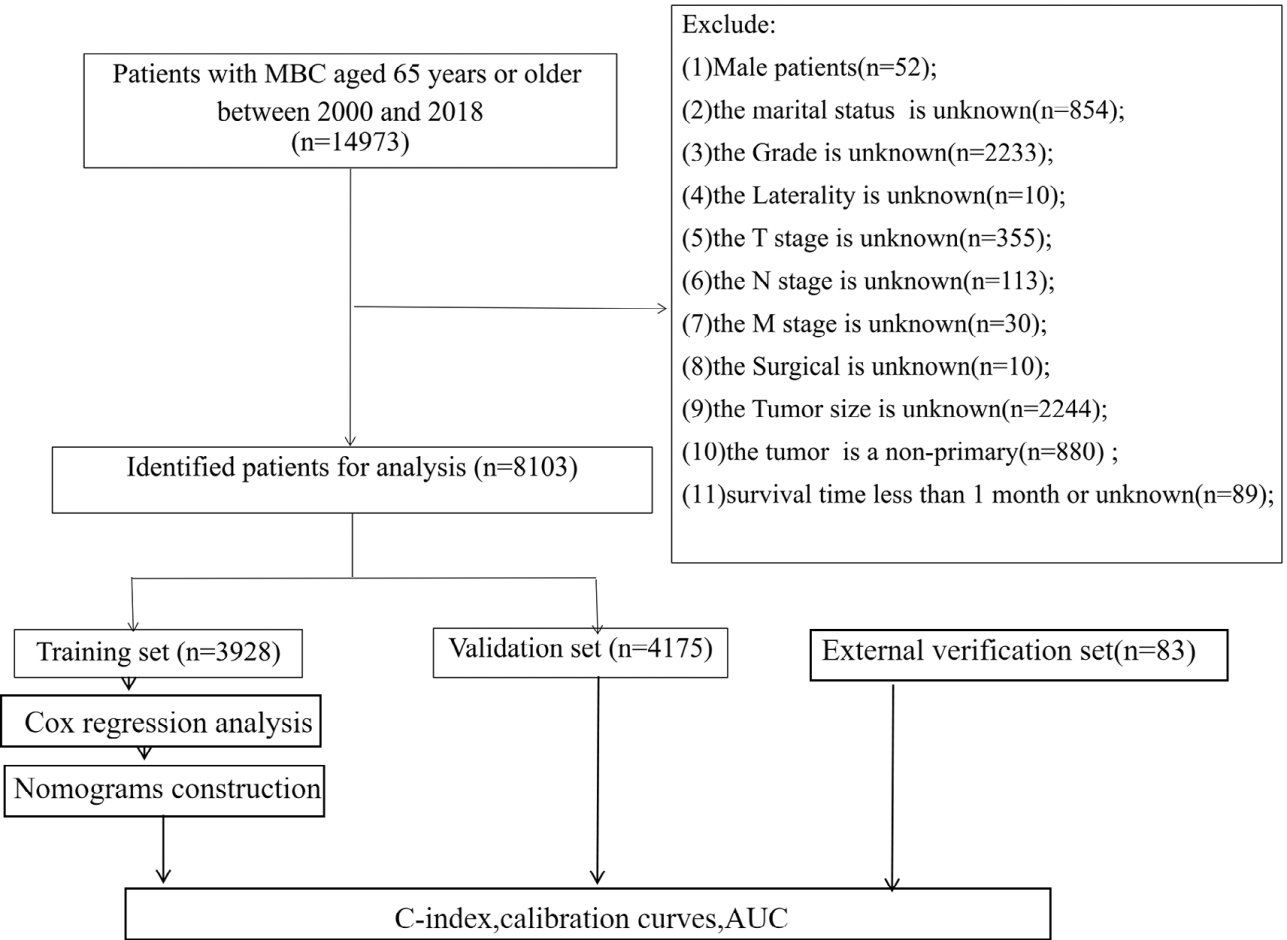
Flowchart for inclusion and exclusion of elderly female patients with MBC.

The breast cancer‐related clinical information contained in the SEER database is the same as the previous study.^16^ The inclusion criteria include the following: (1) Age over 65 (including 65 years old); (2) pathological diagnosis was MBC; (3) female gender. Exclusion criteria: (1) male patients; (2) the marital status is unclear; (3) tumor grade is unclear; (4) laterality is unclear; (5) T stage is unclear; (6) N stage is unclear; (7) M stage is unclear; (8) unknown surgical; (9) unknown tumor size; (10) non‐primary tumor; (11) survival time less than 1 month or ambiguous. Meanwhile, we collected data on elderly MBC patients in Kunming Traditional Chinese Medicine Hospital from 2009 to 2021, which were retrospectively analyzed as an external validation cohort. Identical inclusion and exclusion criteria were ensured (Figure 1). The ethics committee approved the study, and all patients gave informed consent for the study (No. 2022‐lun‐01).

## 3 NOMOGRAM CONSTRUCTION AND VALIDATION

Elderly female MBC patients data was extracted from the SEER database, and cases collected from Kunming Hospital of Traditional Chinese Medicine were used to perform external validation of the model. First, we identified the relevant factors what affecting patients' prognosis. These risk factors were obtained from the training set. Then, using the multivariate Cox proportional regression model screen the independent risk factors related to the prognosis of MBC patients after excluding the bias caused by the single factor. Based on the results of the multivariate regression analysis, we constructed new nomograms for predicting outcomes in elderly female MBC patients. Subsequently, we used calibration curves to test the prediction power of the constructed nomograms at 3, 5 and 8 years. Meanwhile, the consistency index (c‐index) and the area under the curve (AUC), and decision curve analysis (DCA) were performed to fully test the clinical application value of the novel nomograms. To facilitate the use of the researchers, we also made a web version of the nomograms, with links placed in the availability of data and material section.

### 3.1 Statistical analysis

Continuous variables used in this study conformed to normal distribution by testing and were therefore described using means and standard deviations. Differences between groups were analyzed using χ2 test or non‐parametric U test. Categorical variables were described using frequencies for (%), and we used the χ2 test to detect differences between groups. Also, performing Cox risk regression model analysis screen independent risk factors that affect the prognosis of elderly patients with MBC. Differences in patient survival were analyzed through the K‐M curves and log‐rank test. R version 4.2.0 and SPSS 25.0 was used for statistical analysis. The R package used were based on “Survival,” “gg DCA,” “Dyn Nom,” and “RMS”. P‐values less than .05 were considered statistically significant.

## 4 RESULTS

### 4.1 Baseline characteristics of the patients

A total of 8103 female patients diagnosed with MBC and aged over 65 years old (including 65) from SEER database were assigned to two cohorts: the training set (N = 3928) and the validation set (N = 4175). A total of 83 patients from Kunming Hospital of Traditional Chinese Medicine were included into the external verification set patients. The mean age was 76.1 ± 7.27 years; more than 80% were white; 43.7% were married, 36.7% were widowed, divorced, 10.3%, and single, 9.29%. Grade I accounted for 62.7% and grade II accounted for 34.4%. The T stages were T1 (68.9%), T2 (26.2%), T3 (3.92%), and T4 (1.02%), respectively. The N stage was mainly N0 (93.5%), and the M stage was mainly M0 (99.2%). Most of the patients underwent local tumor resection (68.6%), 16.7% underwent a local mass mastectomy, and 11% underwent a radical mastectomy. Patients receiving chemotherapy were 4.26%. The proportion of patients receiving radiotherapy was 43.5%. Not significantly in tumor side, the left and right side were 51% and 49%, respectively. Both ER and PR were mainly positive, representing 95.6% and 88.1%, respectively, whereas HER2 was mainly negative, representing 57.1%. Group differences in the baseline information were not statistically significant, as presented in Table 1.

**TABLE 1:** Clinicopathological characteristics of elderly female patients with MBC.

	ALL	Training set	Validation set	
	N = 8103	N = 3928	N = 4175	p
Age	76.21 (7.27)	76.21 (7.30)	76.1 (7.24)	.652
Race				.673
white	6555 (80.9%)	3172 (80.8%)	3383 (81.0%)	
black	831 (10.3%)	414 (10.5%)	417 (9.99%)	
other	717 (8.85%)	342 (8.71%)	375 (8.98%)	
Marital				.922
Single	753 (9.29%)	360 (9.16%)	393 (9.41%)	
Divorced/Separated	836 (10.3%)	406 (10.3%)	430 (10.3%)	
Widowed	2970 (36.7%)	1430 (36.4%)	1540 (36.9%)	
Married	3544 (43.7%)	1732 (44.1%)	1812 (43.4%)	
Grade				.455
I	5077 (62.7%)	2442 (62.2%)	2635 (63.1%)	
II	2791 (34.4%)	1364 (34.7%)	1427 (34.2%)	
III/IV	235 (2.90%)	122 (3.11%)	113 (2.71%)	
Tumor size	19.7 (19.4)	19.5 (17.7)	19.8 (21.0)	.498
T				.930
T1	5583 (68.9%)	2709 (69.0%)	2874 (68.8%)	
T2	2119 (26.2%)	1021 (26.0%)	1098 (26.3%)	
T3	318 (3.92%)	159 (4.05%)	159 (3.81%)	
T4	83 (1.02%)	39 (0.99%)	44 (1.05%)	
N				.678
N0	7579 (93.5%)	3668 (93.4%)	3911 (93.7%)	
N1	432 (5.33%)	218 (5.55%)	214 (5.13%)	
N2	69 (0.85%)	30 (0.76%)	39 (0.93%)	
N3	23 (0.28%)	12 (0.31%)	11 (0.26%)	
M				.721
M0	8035 (99.2%)	3897 (99.2%)	4138 (99.1%)	
M1	68 (0.84%)	31 (0.79%)	37 (0.89%)	
Surgery				.455
No	293 (3.62%)	136 (3.46%)	157 (3.76%)	
Local tumor excision	5561 (68.6%)	2689 (68.5%)	2872 (68.8%)	
Local mastectomy	1357 (16.7%)	650 (16.5%)	707 (16.9%)	
Radical mastectomy	892 (11.0%)	453 (11.5%)	439 (10.5%)	
Chemotherapy				.057
No	7758 (95.7%)	3743 (95.3%)	4015 (96.2%)	
Yes	345 (4.26%)	185 (4.71%)	160 (3.83%)	
Radiation				.090
No	4579 (56.5%)	2258 (57.5%)	2321 (55.6%)	
Yes	3524 (43.5%)	1670 (42.5%)	1854 (44.4%)	
Laterality				.861
Left	4129 (51.0%)	2006 (51.1%)	2123 (50.9%)	
Right	3974 (49.0%)	1922 (48.9%)	2052 (49.1%)	
ER				.280
Negative	99 (1.22%)	49 (1.25%)	50 (1.20%)	
Positive	7745 (95.6%)	3766 (95.9%)	3979 (95.3%)	
Borderline/Unknown	259 (3.20%)	113 (2.88%)	146 (3.50%)	
PR				.190
Negative	651 (8.03%)	319 (8.12%)	332 (7.95%)	
Positive	7139 (88.1%)	3473 (88.4%)	3666 (87.8%)	
Borderline/Unknown	313 (3.86%)	136 (3.46%)	177 (4.24%)	
HER2				.941
Negative	4623 (57.1%)	2247 (57.2%)	2376 (56.9%)	
Positive	134 (1.65%)	66 (1.68%)	68 (1.63%)	
Borderline/Unknown	3346 (41.3%)	1615 (41.1%)	1731 (41.5%)	
CSS				.883
Dead	388 (4.79%)	190 (4.84%)	198 (4.74%)	
Alive	7715 (95.2%)	3738 (95.2%)	3977 (95.3%)	
OS				.571
Dead	2549 (31.5%)	1248 (31.8%)	1301 (31.2%)	
Alive	5554 (68.5%)	2680 (68.2%)	2874 (68.8%)	
Survival months	68.2 (47.3)	67.5 (47.0)	68.8 (47.5)	.215

### 4.2 Nomograms construction and validation

**FIGURE 2:**
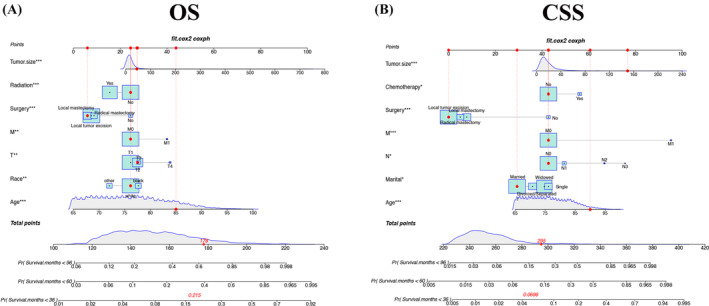
Nomograms predicting 3‐, 5‐, and 8‐year prognosis in elderly female patients with MBC. (A) Nomogram predicting OS in elderly female patients with MBC. (B) Nomogram predicting CSS in elderly female patients with MBC.

The results of univariate and multivariate OS and CSS are presented in Tables 2 and 3. We developed new nomograms by combining the results of independent risk factors that can predict the outcomes in elderly female MBC patients (Figure 2). Radiotherapy, surgical mode, M stage, age, and race were the most important factors affecting patients' OS. Second, tumor size and T stage were also essential factors of OS. Tumor size, surgical mode, M stage, and age were the most important factors for CSS, and second, chemotherapy, N stage, and marital status had less effect on patients' CSS.

**TABLE 2:** Univariate and multivariate analyses of OS in training set.

	Univariate			Multivariate		
	HR	95%CI	P	HR	95%CI	P
Age	1.1	1.09–1.11	<.001	1.085	1.075–1.094	<.001
Race						
white						
black	1.12	0.93–1.34	.236			
other	0.65	0.51–0.83	<.001	0.653	0.51–0.836	.001
Marital						
Single						
Divorced/Separated	0.87	0.67–1.12	.278			
Widowed	1.26	1.03–1.55	.028			
Married	0.67	0.54–0.83	<.001			
T						
T1						
T2	1.61	1.43–1.83	<.001	1.212	1.045–1.406	.011
T3	3.17	2.52–3.98	<.001	1.734	1.201–2.502	.003
T4	4.16	2.83–6.11	<.001			
N						
N0						
N1	1.53	1.23–1.91	<.001			
N2	1.8	1.06–3.05	.029			
N3	2.04	0.85–4.92	.111			
M						
M0						
M1	6.82	4.59–10.14	<.001	4.232	2.749–6.515	<.001
Tumor size	1.01	1.01–1.02	<.001	1.006	1.002–1.011	.005
Surgery						
No						
Local tumor excision	0.21	0.17–0.26	<.001	0.496	0.387–0.637	<.001
Local mastectomy	0.25	0.2–0.33	<.001	0.472	0.362–0.616	<.001
Radical mastectomy	0.29	0.23–0.38	<.001	0.495	0.381–0.644	<.001
Radiation						
No						
Yes	0.48	0.42–0.54	<.001	0.706	0.611–0.815	<.001

**TABLE 3:** Univariate and multivariate analyses of CSS in training set.

	Univariate			Multivariate		
	HR	95%CI	P	HR	95%CI	P
Age	1.06	1.04–1.08	<.001	1.046	1.023–1.07	<.001
Race						
white						
black	1.54	1.02–2.34	.042			
other	0.86	0.49–1.51	.599			
Marital						
Married						
Single	0.72	0.39–1.32	.287			
Divorced/Separated	0.96	0.6–1.52	.856			
Widowed	0.44	0.27–0.73	.001	0.588	0.354–0.975	.04
Grade						
I						
II	1.5	1.11–2.01	.007			
III/IV	1.74	0.85–3.57	.132			
T						
T1						
T2	2.11	1.53–2.9	<.001			
T3	6.62	4.18–10.48	<.001			
T4	11.57	6.02–22.24	<.001			
N						
N0						
N1	3.32	2.19–5.03	<.001			
N2	4.54	1.86–11.07	.001	2.787	1.073–7.241	.035
N3	8.41	2.68–26.38	<.001	3.482	1.039–11.667	.043
M						
M0						
M1	29.25	17.69–48.35	<.001	7.897	4.197–14.858	<.001
Tumor size	1.02	1.02–1.02	<.001	1.01	1.005–1.015	<.001
Surgery						
No						
Local tumor excision	0.07	0.05–0.11	<.001	0.219	0.132–0.363	<.001
Local mastectomy	0.11	0.07–0.18	<.001	0.249	0.145–0.43	<.001
Radical mastectomy	0.14	0.09–0.23	<.001	0.223	0.133–0.373	<.001
Radiation						
No						
Yes	0.45	0.32–0.61	<.001			
Chemotherapy						
No				1.72	1.009–2.933	.046
Yes	2.53	1.61–3.99	<.001			
PR						
Negative						
Positive	0.6	0.4–0.9	.014			
Borderline/Unknown	0.83	0.41–1.68	.605			

**FIGURE 3:**
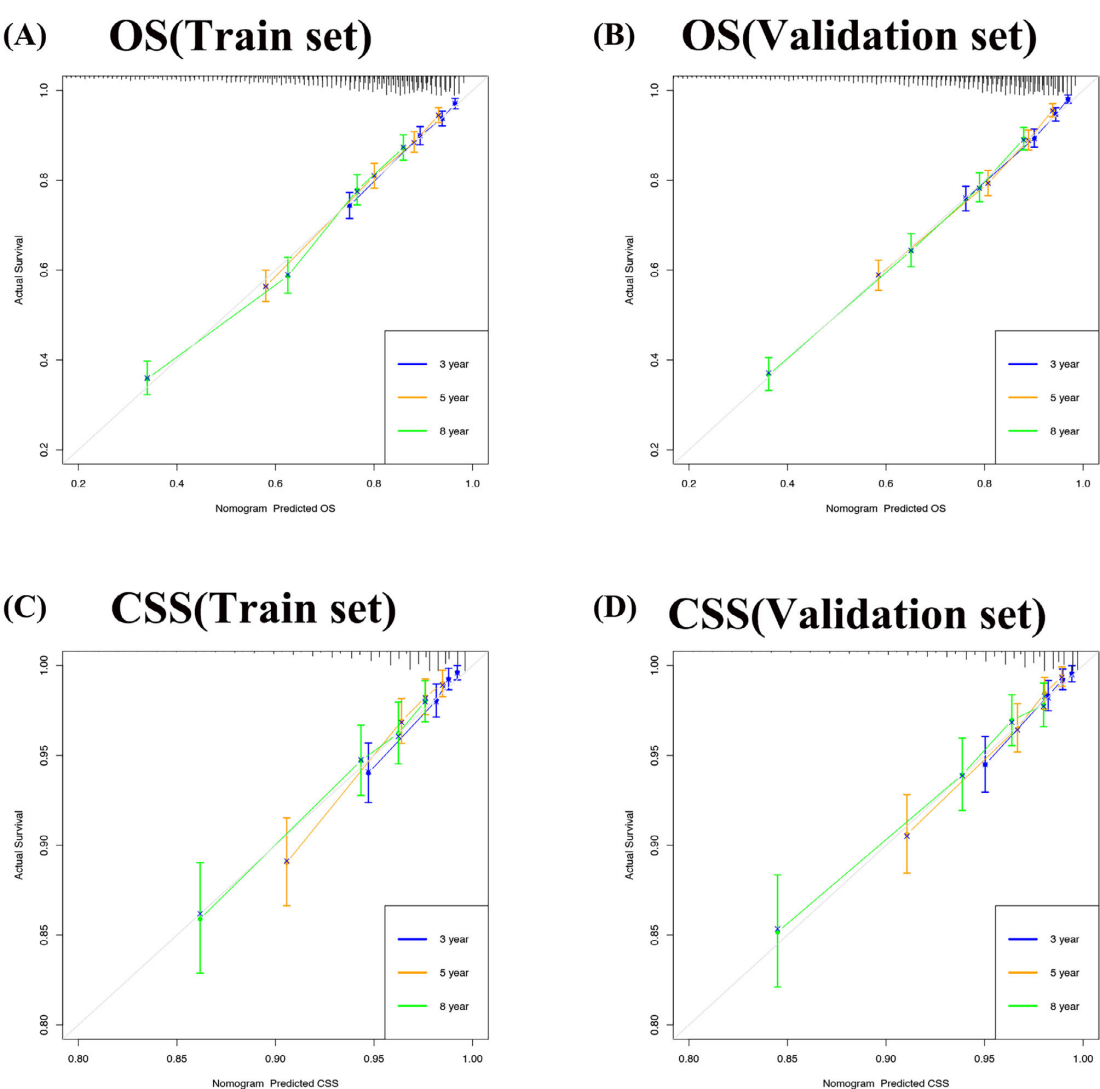
Calibration curves of nomograms predicting 3‐, 5‐, and 8‐year prognosis of elderly female MBC patients. (A) Calibration curves of nomograms on OS in the training set. (B) Calibration curves of nomograms predicting OS in the validation set. (C) Calibration curves of nomograms predicting CSS in the training set. (D) Calibration curves of nomograms predicting CSS in the validation set.

**FIGURE 4:**
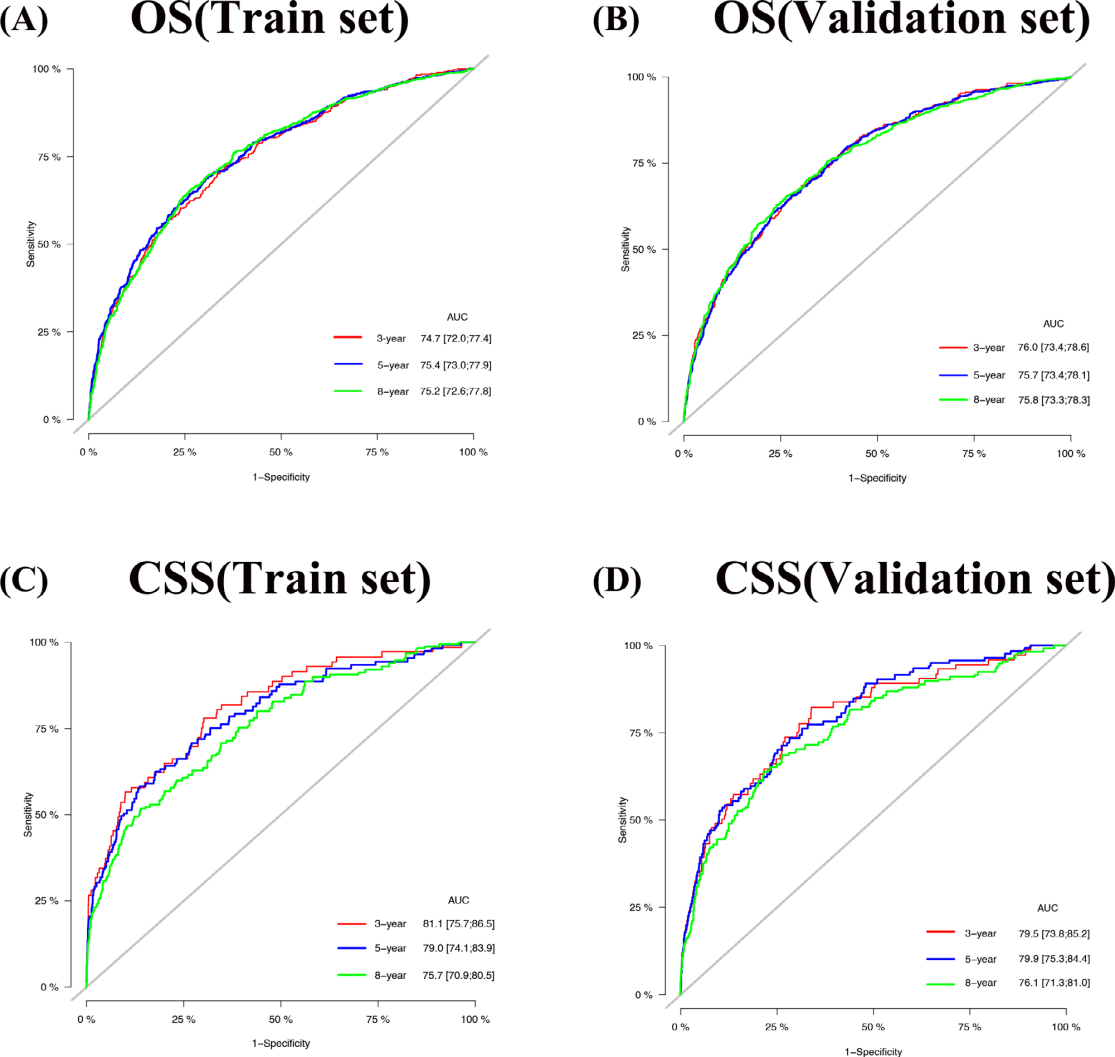
AUC predicts prognosis in elderly female patients with 3‐, 5‐, 8 year MBC. (A) AUC of OS in the training set. (B) AUC of OS in the validation set. (C) AUC of CSS in the training set. (D) AUC of CSS in the validation set.

We validated the accuracy and discrimination of the nomograms. The C‐index for the OS were 0.731 for training set, 0.738 for validation set and 0.809 for the external verification set. The C‐index for CSS were 0.786 in the training set, 0.776 in the validation set, and 0.84 in the external verification set. The C‐index indicates fine recognition of the prediction model of the nomograms. The calibration curve also illustrates the fine prediction accuracy of the nomograms (Figure 3), the Figure S1 showed the calibration curve for OS and CSS in the external verification set, all the results indicated that the nomogram has good accuracy. In the OS training set, the AUC was 74.7, 75.2, 75.4; in the OS validation set, the AUC were 76.0, 75.7, 75.8. In the CSS training set, the AUC was 81.1, 79.0, 75.7; in the CSS validation set, the AUC was 79.5, 79.9, 76.1, respectively (Figure 4). In the external verification set, the AUC was 88.1, 88.6, and 67.7 for OS at 3, 5, and 8 years; the AUC was 82.0, 82.5, and 77.3, respectively for CSS at 3, 5, and 8 years (Figure S2). The above results show that the nomograms we constructed to predict OS and CSS are very discriminative.

### 4.3 Clinical application of nomograms

**FIGURE 5:**
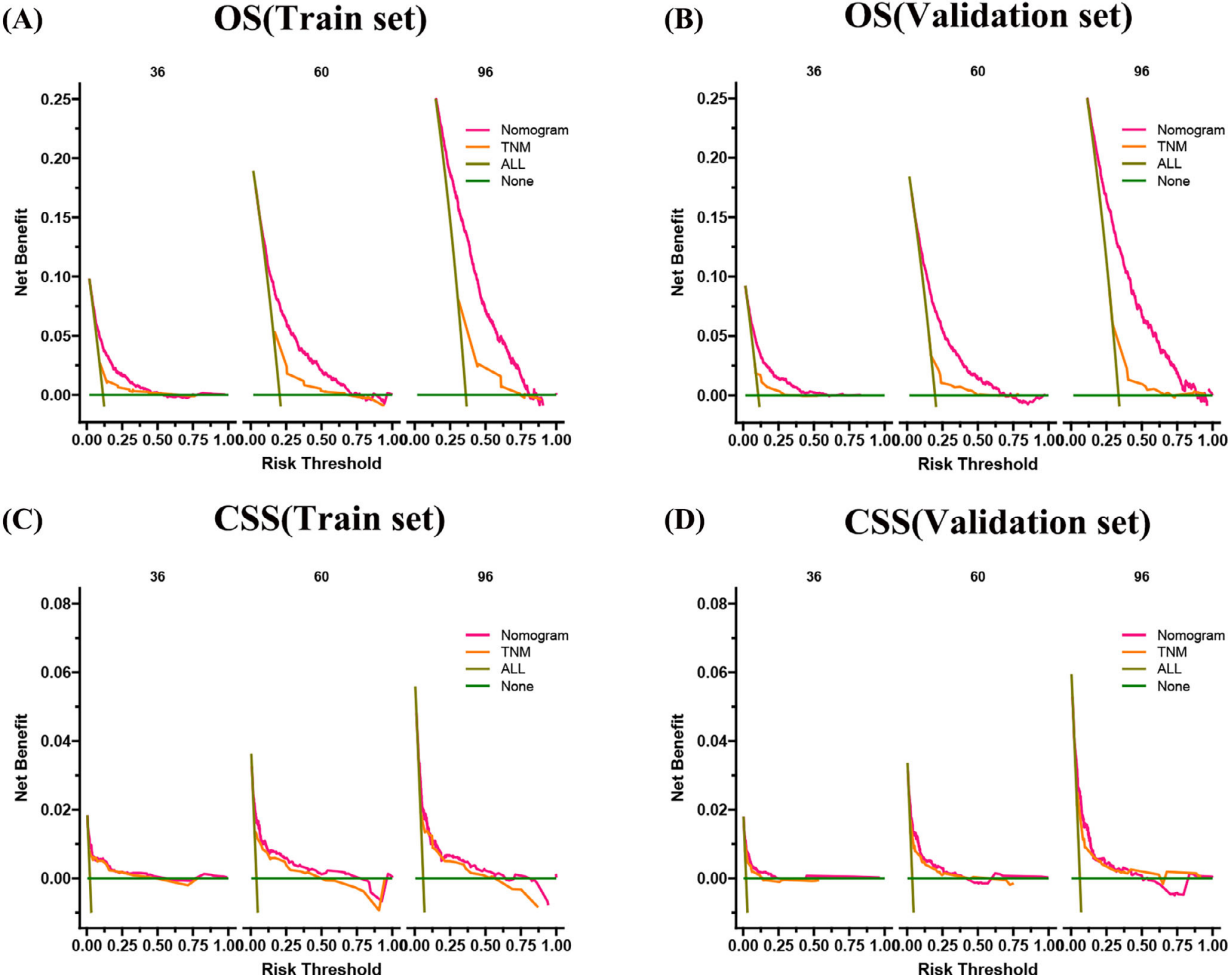
DCA of the nomograms for predicting OS and CSS. (A) DCA of the nomograms for predicting OS in the training set. (B) DCA of the nomograms for predicting OS in the validation set. (C) DCA of the nomograms for predicting CSS in the training set. (D) DCA of the nomograms for predicting CSS in the validation set.

**FIGURE 6:**
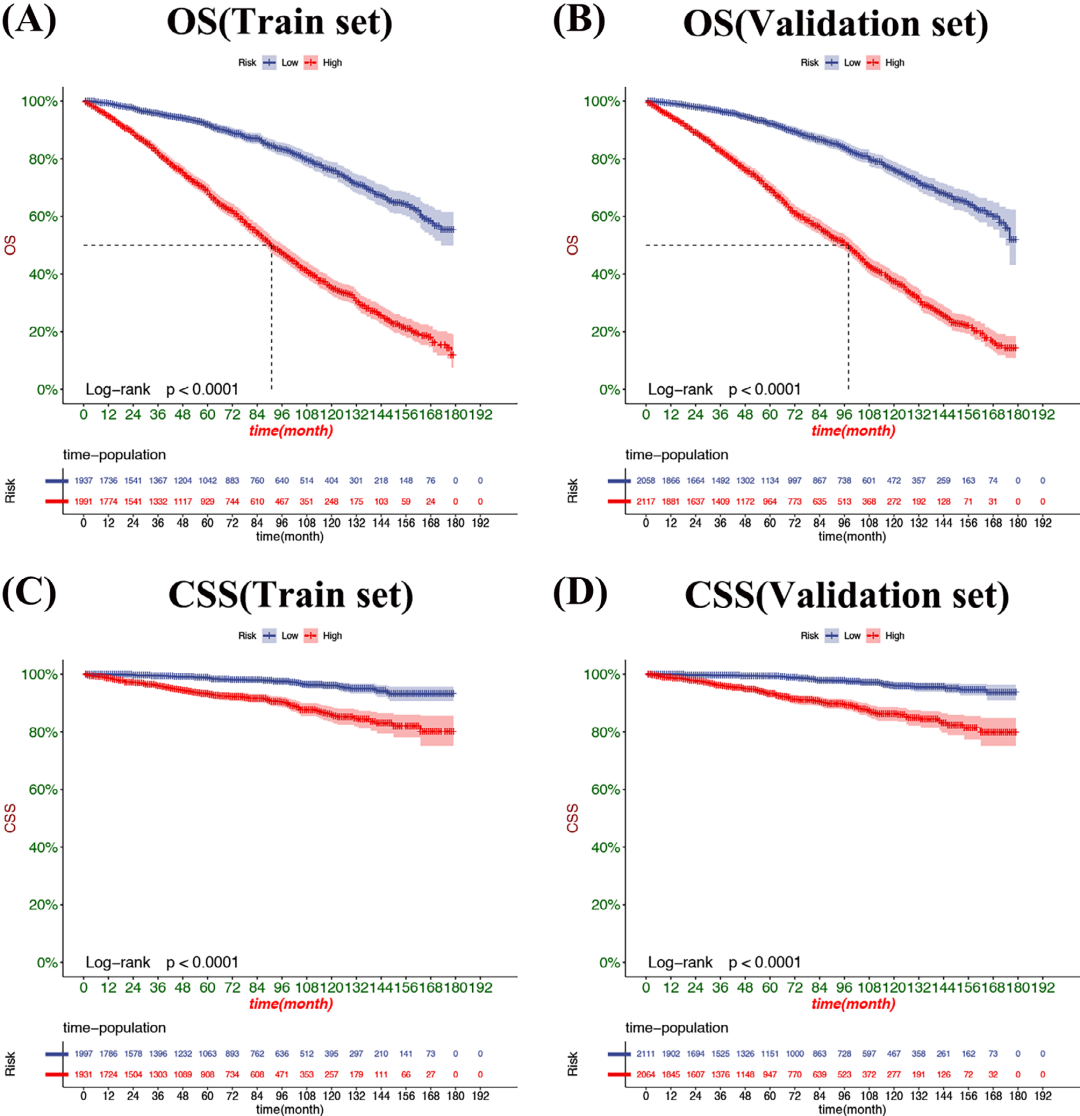
K‐M curves in low‐risk patients and high‐risk group. The OS rates of patients in the high‐risk group are significantly lower than those in the low‐risk group in both the training set (A) and the validation set (B). The same results were observed in the training set (C) and validation set (D) for CSS rate.

**FIGURE 7:**
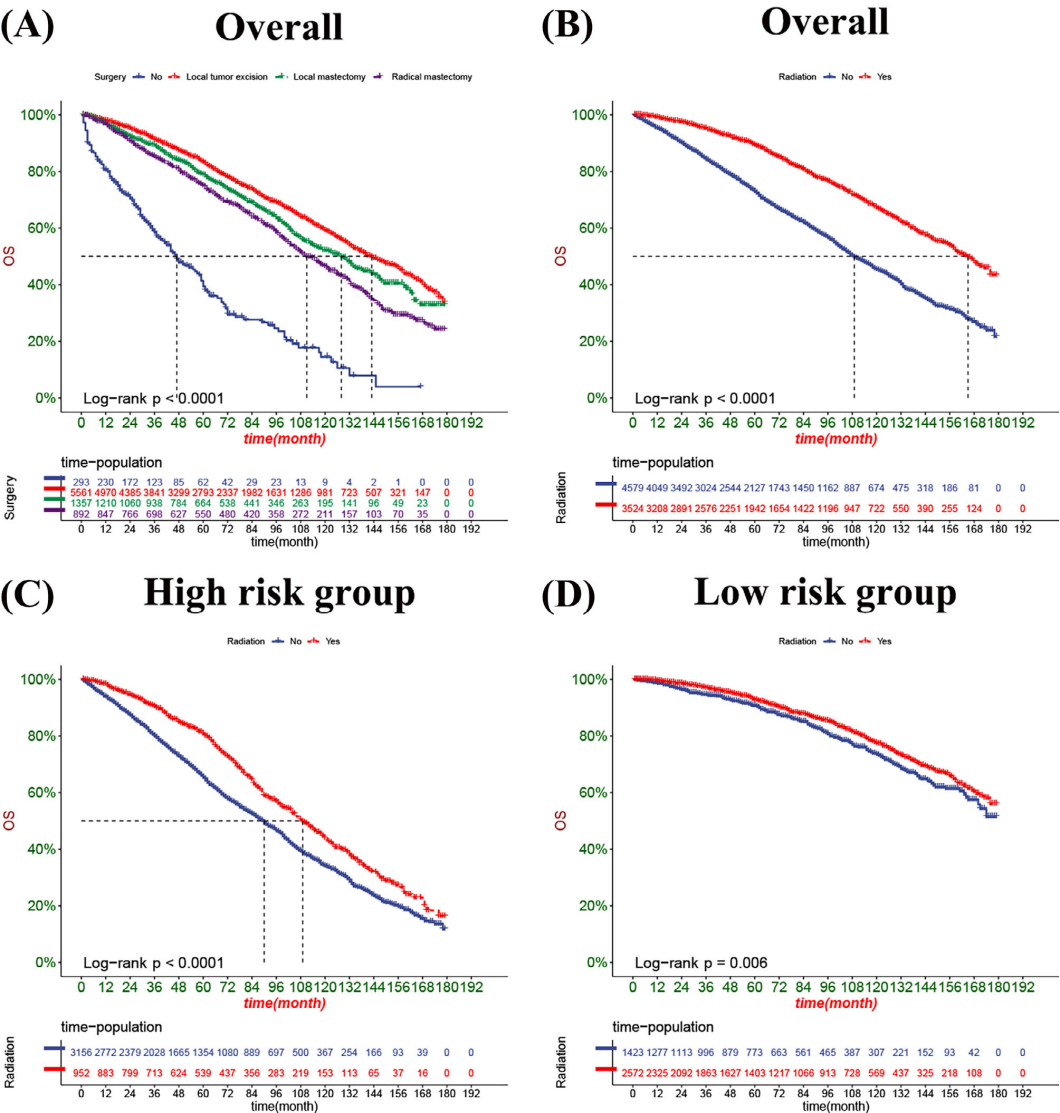
Kaplan–Meier curves of OS rate of patients with different surgery mode and radiotherapy. (A) The OS rate of patients underwent different surgery. (B) The OS rate of patients in patients with or without radiotherapy. (C) The OS rate of patients in the high‐risk group with or without radiotherapy. (D) The OS rate of patients in the low‐risk group with or without radiotherapy.

**FIGURE 8:**
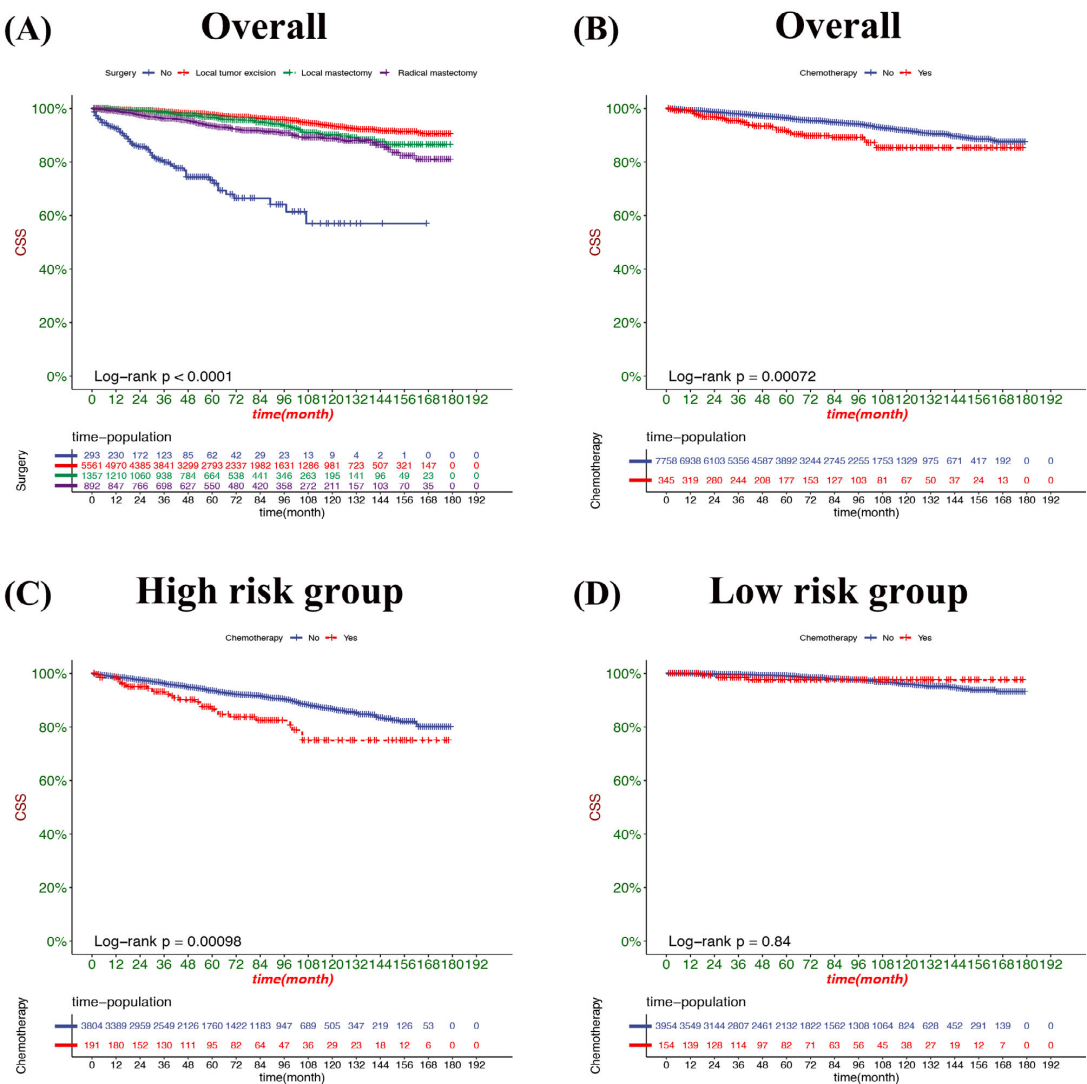
Kaplan–Meier curves of CSS rates in patients with different surgical approaches and chemotherapy. (A) CSS rates in patients with different surgical approaches. (B) CSS rates in patients with and without chemotherapy. (C) OS rates in patients in the high‐risk group with or without chemotherapy. (D) OS rates in patients in the low‐risk group with or without chemotherapy.

Decision curve analysis result presented that the nomogram had fine clinical potential value for the prognosis of elderly female MBC patients (Figure 5), and all were significantly better than the conventional TNM staging. The risk value of each patient was calculated according to the nomogram, and the best cut‐off value was calculated. The patients were divided into high‐risk and low‐risk groups. The K‐M curve showed that the prognosis of the high‐risk group was significantly worse than that of the low‐risk group (Figure 6). The OS of patients without surgery was the lowest, followed by radical mastectomy, and the OS of patients with local tumor resection was the highest. OS was higher in patients treated with radiotherapy, and better survival benefit in high‐risk patients than low‐risk patients (Figure 7). For CSS, the trend of surgical methods was similar to that of OS. Patients with higher CSS were treated by surgery, and those with the highest CSS were treated by local tumor resection. Chemotherapy is a risk factor for prognosis. For the high‐risk group, patients receiving chemotherapy have lower CSS. In contrast, chemotherapy had little effect on CSS in the low‐risk patients (Figure 8).

## 5 DISCUSSION

Although the survival rate of MBC patients is relatively ideal, the survival rate of patients in elderly remains low due to the effects of comorbidities. Second, there is no unified treatment standard due to MBC low morbidity, which may affect patient prognosis. However, many factors affect patient prognosis, including various clinical case factors. Therefore, this study, based on the SEER database with big data, explored univariate and multi factors affecting OS and CSS in MBC patients. We found that age, race, T and M stage, operation method, tumor size and radiotherapy were independent risk factors of OS in patients with MBC. In contrast, age, marital, N and M stage, surgical mode, tumor size and chemotherapy were play an important role in CSS.

Age is strongly associated with the onset of many cancers, for example prostate cancer, bladder cancer, and other tumors.^17^, ^18^ The incidence of BC is also age‐related, and MCB is a disease that occurs in elderly women, with a mean age of patients significantly more significant than the other histological subtypes.^19^ Meanwhile, a clinical trial evaluating the influence of age on mortality 10 years after diagnosis found that older of age at diagnosis, the increased mortality in BC.^20^ Our study found that for female MBC patients over 65 years old, age affected both the OS and CSS and the older the age, the worse the patient prognosis, in line with the conclusions of previous studies.^21^ The reasons may be as follows: First, most elderly patients have combined comorbidities. Moreover, due to their poor functional status, most elderly patients do not choose an active treatment, resulting in a relatively poor prognosis.^22^

Marital status is also a risk factor associated with many cancer outcomes. Most studies have found that married status favors prognosis for cancer patients; for example, in colon cancer, divorced/separated/widowed patients are associated with higher mortality when compared with married patients.^23^ Studies have shown that unmarried or widowed women have a e a higher diagnostic risk and worse survival outcomes than married women. Therefore, more attention should be given to single female BC patients.^24^ The clinical nomogram constructed by Fu et al. to predict the survival outcome of early MBC showed a better CSS with married patients.^12^ Our study divided the marital status of MBC patients into four types, including single, divorced, widowed as well as married. For elderly patients with MBC, married patients have a better CSS, with little difference in survival among single, divorced, and widowed patients. Considering that married patients can get more financial support and care and more emotional comfort.

Race is strongly associated with the incidence and mortality of BC patients. In recent years, the mortality of white patients has decreased due to early detection and improved treatment options. But overall, cancer incidence in African Americans and Hispanic Americans continues to rise.^25^ The predictive model constructed by Pan et al. showed that race was an independent risk factor affecting the OS of patients with inflammatory BC.^8^ Min et al. found that race can affect regional lymph node metastasis risk in patients with T1‐2 MBC.^26^ The predictive model for MBC constructed by Zhu et al. showed that race was an independent risk factor for OS in MBC patients.^11^ Our results also showed that ethnicity was strongly related to OS in older female MBC patients but it had no effect on the CSS in MBC patients.

TNM stage is the traditional way to evaluate tumor prognosis. The prognosis of most cancer patients is closely related to the TNM stage. Our study found that the TNM stage also seriously affected OS and CSS in older female MBC patients. Overall, the higher the TNM stage, the worse the prognosis, and previous studies showed consistent.^11^, ^12^ In addition, tumor size is also a key factor which affect the survival of cancer patients. However, the AJCC staging system suggested that tumor size may not be an important prognostic factor for MBC, because the vast majority of the tumor volume is mucin.^27^ For some time in the past, patients with tumor size >2 cm who should receive adjuvant chemotherapy was written into the NCCN guidelines. However, at this stage, the guidelines have been revised; regardless of this breast cancer patient's T‐stage at any stage, patients with lymph node metastases are considered to have an indication for chemotherapy as long as they have.^28^ Therefore, tumor size is crucial for the choice of treatment regimen for patients with MBC. However, some scholars have pointed out that tumor size may not be accurately measured, which may lead to bias in the judgment of prognosis.^29^ However, the nomogram constructed by Wu et al. shows that tumor size is an independent risk factor for predicting the probability of lymph node metastasis in MBC patients, and the larger the tumor, the higher the risk of lymph node metastasis.^30^ For elderly MBC patients, our results also show that tumor size for patients with OS and CSS has a significant impact; the larger the tumor, the lower the OS and CSS, considering the bigger the tumor, the more difficult the operation and for elderly patients, due to a variety of comorbidities, the larger the tumor, the worse the tolerance of patients.

At present, for the treatment of BC. Surgical methods for BC include local tumor resection, partial mastectomy, and radical mastectomy. However, for MBC, there is no unified and standardized treatment plan due to its low incidence, and its treatment method refers to invasive BC. The current mainstream treatment strategy is surgery combined with adjuvant therapy, but its survival benefit is not clear. Recently, local treatment for stage T1‐2 MBC has recommended breast preservation, which has a better prognosis than radical mastectomy.^31^ The study by Kaisu Ojala et al. showed that OS and elderly breast cancer patients undergoing surgery had better CSS, suggesting that surgical treatment also seems to be safe in the elderly patient population.^32^ The study by Hu et al. showed that surgery is a protective factor for the overall survival of MBC patients.^21^ This study found that most of the MBC patients received surgical treatment, and the OS and CSS of MBC patients benefited from surgical treatment, the results of the KM curve analysis showed that patients undergoing local resection of the tumor had the best prognosis, followed by patients treated with local mastectomy, while patients undergoing radical mastectomy had a worse prognosis than the first two surgical procedures. Considering that elderly MBC patients are prone to complicated comorbidities, radical surgery is poorly tolerated.

Radiation therapy is one of the most important therapeutic measures for many cancer patients. A randomized controlled trial found that the ipsilateral breast tumor recurrence rate without radiotherapy was relatively high for in patients ≥65 with early BC compared to women receiving radiotherapy.^33^ But it is increasingly recognized that RT has little benefit in women aged ≥65 who are estrogen receptor positive and with a tumor size ≤3 cm.^34^ While the randomized clinical trial conducted by Jayant S Vaidya et al. showed that It indicates that the immediate single‐dose of target‐iort for risk adaptation during tumor resection is an effective alternative to EBRT in early‐stage BC patients.^35^ Our study found that radiotherapy favored the OS of elderly female MBC patients, and after dividing the patients into different groups, high‐risk patients benefited more from RT, while it had little effect on OS in low‐risk patients. Considering that low‐risk patients can achieve better survival through surgical treatment, radiotherapy has little benefit for low‐risk patients. Moreover, only a few of high‐risk patients receiving RT may bias the results.

Chemotherapy is also one of the adjuvant treatment measures for most cancers. In clinical practice, with an increasing number of patients with triple‐negative BC with TNBC and her2‐positive BC, neoadjuvant chemotherapy has become the preferred therapeutic strategy.^36^ However, the literature has reported that the low chemotherapy efficacy of MBC patients is mainly due to the high proportion of mucus to the total MBC cell volume, forming a large mucin pool, leading to inconsistent clinical or imaging evaluation of chemotherapy efficacy and with mucinous carcinoma pathology.^37^ Despite studies showing that chemotherapy is effective in eliminating malignant cells. However, the mucin pool still exists.^38^ This study found that a very small number of elderly female MBC patients received chemotherapy, and chemotherapy does have no survival benefit in elderly patients with MBC, especially for patients with high risk; chemotherapy is more harmful to CSS, considering the side effects of chemotherapy and the low‐risk patients. However, for low‐risk patients, chemotherapy has little impact on their CSS.

This study explored the influencing factors associated with OS and CSS in elderly female patients with MBC using the SEER database, and we successfully developed nomograms of OS and CSS. However, this study still has some limitations. For example, the HER2 indicators in breast cancer patients are extremely important for patient prognosis, but the SEER database did not begin to include these indicators until 2010, so many breast cancer patients lack relevant data, which will bias the results. Second, this study was retrospective, which would confer selection bias. However, it have included many important key variables and was internally validated, so it will not be greatly biased. Moreover, the KM curve analysis found that the 3‐year, 5‐year, and 8‐year OS was significantly lower than the CSS in both the high‐risk and low‐risk groups. This thus reflects a higher proportion of non‐cancer‐specific deaths in older female MBC patients. Follow‐up studies continuing to explore the risk factors associated with noncancer‐specific death in older MBC patients may be of great clinical value.

## 6 CONCLUSIONS

This study explored the independent risk factors for OS and CSS in elderly female MBC patients, we found that race, age, T stage, M stage, radiotherapy, surgery, and tumor size were independent risk factors for OS in elderly MBC patients. Age, marital status, NM stage, surgery, tumor size, and chemotherapy were independent risk factors for CSS. Independent risk factors were screened according to COX multifactorial, and based on these factors, nomograms were constructed to predict OS and CSS in elderly MBC patients. Through a series of validations, our study confirmed that these new nomograms have better accuracy compared with previous studies. It can provide effective help to clinicians when they conduct the diagnosis and treatment of elderly MBC patients. Our study also found that surgery and radiotherapy favored survival in elderly female MBC patients, while chemotherapy had little survival benefit. Moreover, this study found that the OS of elderly female MBC patients was significantly lower than the CSS, suggesting a higher proportion of non‐cancer‐specific deaths, so the follow‐up studies will continue to explore the non‐cancer‐specific death‐related risk factors.

## AUTHOR CONTRIBUTIONS

**Zhaoxia Zhang:** Conceptualization (equal); data curation (equal); methodology (equal); writing – original draft (equal). **Chenghao Zhanghuang:** Conceptualization (equal); validation (equal). **Qian Cai:** Conceptualization (equal); visualization (equal). **Guangye Song:** Data curation (equal). **Quan Wang:** Funding acquisition (equal); methodology (equal). **Yue Tang:** Data curation (equal). **Hongbo Li:** Funding acquisition (equal); writing – review and editing (equal).

## FUNDING INFORMATION

This study was supported by Chongqing Science and Health Joint Project (No. 2023MSXM070) the youth program of clinical medicine research of National Children's Health and Disease Clinical Medicine Research Centre (No. NCRCCHD‐2021‐YP‐17). The funding bodies played no role in the study's design and collection, analysis and interpretation of data, and writing the manuscript.

## CONFLICT OF INTEREST STATEMENT

The authors declare that they have no competing interests.

## ETHICS STATEMENT AND CONSENT TO PARTICIPATE

The data from the SEER database is public and anonymous, so ethical approval and informed consent does not require. The data in the external verification set from Kunming Hospital of Traditional Chinese Medicine. Informed consent was obtained from all patients before enrollment, and this study was approved by the Ethics Committee of Kunming Hospital of Traditional Chinese Medicine.

## Supporting information


**Figure S1.** Externally validated calibration curves, predicted 3‐, 5‐, 8 year OS and CSS. A: External validation focused on predicting patient OS. B: External validation focused on predicting patient CSS.

**Figure S2.** AUC for predicting 3‐, 5‐, and 8‐year OS and CSS in the external verification set. A: The AUC at 3‐, 5‐, and 8‐year for OS in the external verification set. B: The AUC at 3‐, 5‐, and 8‐year for CSS in the external verification set.

## Data Availability

The SEER data analyzed in this study is available at https://seer.Cancer.gov/. Nomogram link of the web version::https://cssnomogram.shinyapps.io/DynNomapp/ and https://osnomogram1.shinyapps.io/DynNomapp/.

## References

[1] Bray F , Ferlay J , Soerjomataram I , Siegel RL , Torre LA , Jemal A . Global cancer statistics 2018: GLOBOCAN estimates of incidence and mortality worldwide for 36 cancers in 185 countries. CA Cancer J Clin. 2018;68(6):394‐424. doi:10.3322/caac.21492 30207593

[2] Siegel RL , Miller KD , Fuchs HE , Jemal A . Cancer Statistics, 2021. CA Cancer J Clin. 2021;71(1):7‐33. doi:10.3322/caac.21654 33433946

[3] Anderson WF , Chu KC , Chang S , Sherman ME . Comparison of age‐specific incidence rate patterns for different histopathologic types of breast carcinoma. Cancer Epidemiol Biomarkers Prev. 2004;13(7):1128‐1135. 15247123

[4] Li CI . Risk of mortality by histologic type of breast cancer in the United States. Horm Cancer. 2010;1(3):156‐165. doi:10.1007/s12672-010-0016-8 21761358 PMC10357995

[5] Bae SY , Choi MY , Cho DH , Lee JE , Nam SJ , Yang JH . Mucinous carcinoma of the breast in comparison with invasive ductal carcinoma: clinicopathologic characteristics and prognosis. J Breast Cancer. 2011;14(4):308‐313. doi:10.4048/jbc.2011.14.4.308 22323918 PMC3268928

[6] Komaki K , Sakamoto G , Sugano H , Morimoto T , Monden Y . Mucinous carcinoma of the breast in Japan. A prognostic analysis based on morphologic features. Cancer. 1988;61(5):989‐996. doi:10.1002/1097-0142(19880301)61:5<989::aid-cncr2820610522>3.0.co;2-e 2827884

[7] Corso G , Maisonneuve P , Massari G , et al. Validation of a novel nomogram for prediction of local relapse after surgery for invasive breast carcinoma. Ann Surg Oncol. 2020;27(6):1864‐1874. doi:10.1245/s10434-019-08160-7 31965372 PMC7523878

[8] Pan X , Yang W , Chen Y , Tong L , Li C , Li H . Nomogram for predicting the overall survival of patients with inflammatory breast cancer: a SEER‐based study. Breast. 2019;47:56‐61. doi:10.1016/j.breast.2019.05.015 31351202

[9] Kim SY , Cho N , Choi Y , et al. Factors affecting pathologic complete response following Neoadjuvant chemotherapy in breast cancer: development and validation of a predictive nomogram. Radiology. 2021;299(2):290‐300. doi:10.1148/radiol.2021203871 33754824

[10] Beato Tortajada I , Albiach CF , Macias VM . Nomogram for the personalisation of radiotherapy treatments in breast cancer patients. Breast. 2021;60:255‐262. doi:10.1016/j.breast.2021.11.004 34808437 PMC8609093

[11] Zhu X , Li Y , Liu F , et al. Construction of a prognostic nomogram model for patients with mucinous breast cancer. J Healthc Eng. 2022;2022:1230812. doi:10.1155/2022/1230812 35368964 PMC8967531

[12] Fu J , Wu L , Jiang M , et al. Clinical nomogram for predicting survival outcomes in early mucinous breast cancer. PLoS One. 2016;11(10):e0164921. doi:10.1371/journal.pone.0164921 27760180 PMC5070827

[13] Marrazzo E , Frusone F , Milana F , et al. Mucinous breast cancer: a narrative review of the literature and a retrospective tertiary single‐centre analysis. Breast. 2020;49:87‐92. doi:10.1016/j.breast.2019.11.002 31783314 PMC7375663

[14] Kathleen N , Lohr E . In: Lohr KN , ed. Medicare: A Strategy for Quality Assurance, in Medicare: A Strategy for Quality Assurance. Washington (DC); 1990.

[15] WHO j Proposed working definition of an older person in Africa for the MDS Project. 2017.

[16] Lin S , Liu C , Tao Z , Zhang J , Hu X . Clinicopathological characteristics and survival outcomes in breast carcinosarcoma: a SEER population‐based study. Breast. 2020;49:157‐164. doi:10.1016/j.breast.2019.11.008 31812891 PMC7375547

[17] Dall'Era MA , de Vere‐White R , Rodriguez D , Cress R . Changing incidence of metastatic prostate cancer by race and age, 1988‐2015. Eur Urol Focus. 2019;5(6):1014‐1021. doi:10.1016/j.euf.2018.04.016 29735368

[18] Plouvier SD , Bonnal JL , Machuron F , et al. Impact of age on bladder cancer management practices: a general population study. Acta Oncol. 2020;59(4):462‐466. doi:10.1080/0284186X.2020.1723164 32043407

[19] Zhang L , Jia N , Han L , Yang L , Xu W , Chen W . Comparative analysis of imaging and pathology features of mucinous carcinoma of the breast. Clin Breast Cancer. 2015;15(2):e147‐e154. doi:10.1016/j.clbc.2014.11.005 25523373

[20] Derks MGM , Bastiaannet E , van de Water W , et al. Impact of age on breast cancer mortality and competing causes of death at 10 years follow‐up in the adjuvant TEAM trial. Eur J Cancer. 2018;99:1‐8. doi:10.1016/j.ejca.2018.04.009 29885375

[21] Hu T , Huang J , Fang K . Overall survival in patients with mucinous carcinoma of breast: a population‐based study. Int J Gen Med. 2021;14:9991‐10001. doi:10.2147/IJGM.S343137 34984023 PMC8702984

[22] de Boer AZ , Bastiaannet E , Putter H , et al. Prediction of other‐cause mortality in older patients with breast cancer using comorbidity. Cancers. 2021;13(7):1627‐1640. doi:10.3390/cancers13071627 33915755 PMC8036543

[23] Lee S , Ma C , Zhang S , et al. Marital status, living arrangement, and cancer recurrence and survival in patients with stage III colon cancer: Findings from CALGB 89803 (Alliance). Oncologist. 2022;27(6):e494‐e505. doi:10.1093/oncolo/oyab070 35641198 PMC9177101

[24] Yuan R , Zhang C , Li Q , Ji M , He N . The impact of marital status on stage at diagnosis and survival of female patients with breast and gynecologic cancers: a meta‐analysis. Gynecol Oncol. 2021;162(3):778‐787. doi:10.1016/j.ygyno.2021.06.008 34140180

[25] Yedjou CG , Sims JN , Miele L , et al. Health and racial disparity in breast cancer. Adv Exp Med Biol. 2019;1152:31‐49. doi:10.1007/978-3-030-20301-6_3 31456178 PMC6941147

[26] Min Y , Wei X , Chen H , Xiang K , Yin G , Feng Y . Identifying Clinicopathological risk factors of the regional lymph node metastasis in patients with T1‐2 mucinous breast cancer: a population‐based study. J Oncol. 2021;2021:3866907. doi:10.1155/2021/3866907 34306075 PMC8285172

[27] Komenaka IK , El‐Tamer MB , Troxel A , et al. Pure mucinous carcinoma of the breast. Am J Surg. 2004;187(4):528‐532. doi:10.1016/j.amjsurg.2003.12.039 15041505

[28] Gradishar WJ , Moran MS , Abraham J , et al. NCCN Guidelines(R) Insights: Breast Cancer, Version 4.2021. Natl Compr Canc Netw. 2021;19(5):484‐493. doi:10.6004/jnccn.2021.0023 34794122

[29] Ranade A , Batra R , Sandhu G , Chitale RA , Balderacchi J . Clinicopathological evaluation of 100 cases of mucinous carcinoma of breast with emphasis on axillary staging and special reference to a micropapillary pattern. J Clin Pathol. 2010;63(12):1043‐1047. doi:10.1136/jcp.2010.082495 20962055

[30] Wu SL , Gai JD , Yu XM , Mao X , Jin F . A novel nomogram and risk classification system for predicting lymph node metastasis of breast mucinous carcinoma: a SEER‐based study. Cancer Med. 2022;11:4767‐4783. doi:10.1002/cam4.4804 35599552 PMC9761057

[31] Yu P , Liu P , Zou Y , et al. Breast‐conserving therapy shows better prognosis in mucinous breast carcinoma compared with mastectomy: a SEER population‐based study. Cancer Med. 2020;9(15):5381‐5391. doi:10.1002/cam4.3202 32515157 PMC7402828

[32] Ojala K , Meretoja TJ , Mattson J , Leidenius MHK . Surgical treatment and prognosis of breast cancer in elderly–a population‐based study. Eur J Surg Oncol. 2019;45(6):956‐962. doi:10.1016/j.ejso.2019.01.019 30691722

[33] Kunkler IH , Williams LJ , Jack WJ , Cameron DA , Dixon JM , P I investigators . Breast‐conserving surgery with or without irradiation in women aged 65 years or older with early breast cancer (PRIME II): a randomised controlled trial. Lancet Oncol. 2015;16(3):266‐273. doi:10.1016/S1470-2045(14)71221-5 25637340

[34] Hughes KS , Schnaper LA . Can older women with early breast cancer avoid radiation? Lancet Oncol. 2015;16(3):235‐237. doi:10.1016/S1470-2045(15)70014-8 25637341

[35] Vaidya JS , Bulsara M , Baum M , et al. Long term survival and local control outcomes from single dose targeted intraoperative radiotherapy during lumpectomy (TARGIT‐IORT) for early breast cancer: TARGIT‐A randomised clinical trial. BMJ. 2020;370:m2836. doi:10.1136/bmj.m2836 32816842 PMC7500441

[36] Montemurro F , Nuzzolese I , Ponzone R . Neoadjuvant or adjuvant chemotherapy in early breast cancer? Expert Opin Pharmacother. 2020;21(9):1071‐1082. doi:10.1080/14656566.2020.1746273 32237920

[37] Lannigan AK , Going JJ , Weiler‐Mithoff E , Cooke TG . Mucinous breast carcinoma. Breast. 2002;11(4):359‐361. doi:10.1054/brst.2002.0417 14965697

[38] Didonato R , Shapiro N , Koenigsberg T , D'Alfonso T , Jaffer S , Fineberg S . Invasive mucinous carcinoma of the breast and response patterns after neoadjuvant chemotherapy (NAC). Histopathology. 2018;72(6):965‐973. doi:10.1111/his.13451 29220097

