# Supplementary figures and images for: Nomogram for predicting outcomes in elderly women with mucinous breast cancer: A retrospective study combined with external validation in southwest China
Source: Cancer Rep (Hoboken). 2024 Jul 25;7(7):e2112. doi: 10.1002/cnr2.2112 (PMC11270325; doi:10.1002/cnr2.2112)

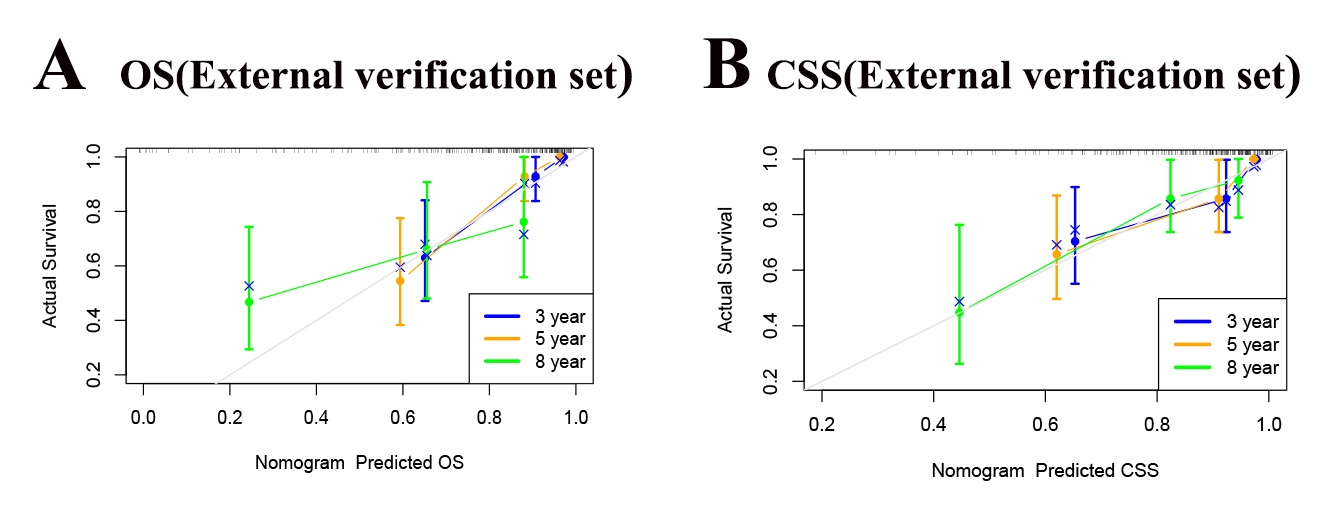

Supplement: Supplementary file 1 — Figure S1. Externally validated calibration curves, predicted 3‐, 5‐, 8 year OS and CSS. A: External validation focused on predicting patient OS. B: External validation focused on predicting patient CSS. [file CNR2-7-e2112-s002.tif]

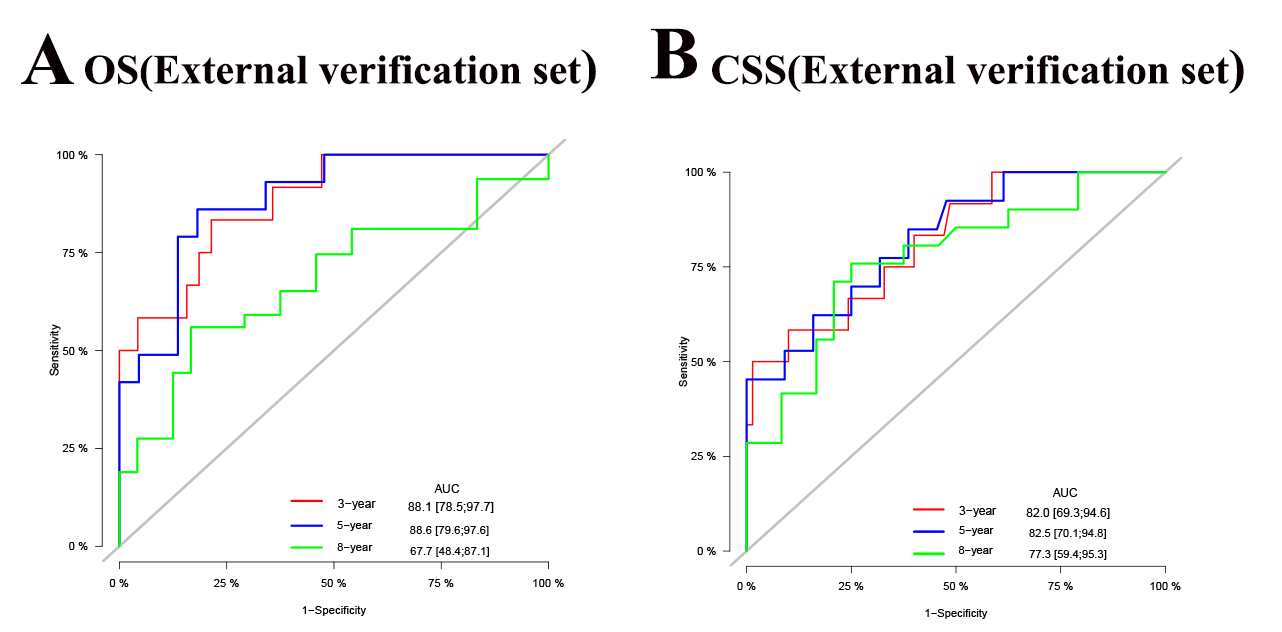

Supplement: Supplementary file 2 — Figure S2. AUC for predicting 3‐, 5‐, and 8‐year OS and CSS in the external verification set. A: The AUC at 3‐, 5‐, and 8‐year for OS in the external verification set. B: The AUC at 3‐, 5‐, and 8‐year for CSS in the external verification set. [file CNR2-7-e2112-s001.tif]
